# Indwelling central venous catheter infection with *Chryseobacterium shandongense* – successful eradication in a 5-year-old with cystic fibrosis

**DOI:** 10.1099/acmi.0.000700.v3

**Published:** 2023-12-05

**Authors:** Anthony Rowan, Tiarnan Fallon Verbruggen, Nuala H. O'Connell, Patrick J. Stapleton, Colum P. Dunne, Barry Linnane, Daryl Butler

**Affiliations:** ^1^​ Department of Paediatrics, University Hospital Limerick, Dooradoyle, Limerick, Ireland; ^2^​ Department of Microbiology, University Hospital Limerick, Dooradoyle, Limerick, Ireland; ^3^​ School of Medicine and Centre for Interventions in Infection, Inflammation and Immunity (4i), University of Limerick, Limerick, Ireland

**Keywords:** *Chryseobacterium shandongense*, cystic fibrosis, port-a-cath infection

## Abstract

**Introduction.:**

*
Chryseobacterium shandongense* is a Gram-negative *Flavobacterium* bacillus with intrinsic multidrug-resistant properties.

**Case Presentation.:**

Herein, we present the first case report of human *C. shandongense* infection, relating to an implantable portal and catheter (port-a-cath) central line in a 5-year-old female with cystic fibrosis. The infection was identified using a Bruker MALDI-TOF Biotyper with BDAL (v12) of blood, which was cultured due to pyrexia and rigour following port-a-cath access. This report details the effective eradication of *C. shandongense* infection from the port-a-cath device using initial empirical gentamicin followed by targeted ciprofloxacin locks and systemic antibiotics.

**Conclusion.:**

We demonstrated successful eradication of *C. shandongense* from a port-a-cath device, including the minimum inhibitory concentrations (MICs) required in this case. The result was eradication of central access infection, preventing progression to bacteraemia/septicaemia and preserving central access in a child with cystic fibrosis and established respiratory disease.

## Data Summary

All data generated or analysed are included in this article.

## Introduction

There are limited reports of *Chryseobacterium* spp. infection and human pathogenesis [1]. The reports of *Chryseobacterium* spp. primarily describe *Chryseobacterium indologenes* (previously *Elizabethkingia meningoseptica *) infections of young infants, nosocomial infection from indwelling devices and infections in immunosuppressed patients [1]. Moreover, to the best of our knowledge, there are no reported cases of *Chryseobacterium shandongense* causing adult or paediatric infection and, specifically, there are no reported infections of cystic fibrosis (CF) patients. *C. shandongense* is a Gram-negative bacillus, a non-motile, non-spore-forming and non-fermentative flavobacterium member of the family W *eeksellaceae *. The human microbiome is not known to include *C. shandongense *, which is typically detected in freshwater and soil sources [2]. Infections associated with *Chryseobacterium* spp. are described, predominately, as nosocomial from indwelling devices and in immunosuppressed patients with associated high mortality secondary to inherent multidrug resistance [1].

This case report describes the successful diagnosis and eradication of *C. shandongense* infection of an implantable portal and catheter (port-a-cath) device in a 5-year-old female patient with CF through the use of antibiotic locks and systemic antibiotics.

## Case presentation

A 5-year-old pancreatic-insufficient female with non-modulator-amenable (genotype 1717/1G>A/Q439X) CF required port-a-cath insertion in July 2022 due to established severe upper lobe bronchiectasis requiring regular intravenous antibiotic therapy. Prior sputum cultures demonstrated *Streptococcus pseudopneumoniae* (January 2018), *Moraxella catarrhalis* (March 2018), *Haemophilus influenzae* (May 2019, March 2023), *Pseudomonas putida* (June 2017, March 2018), *Staphylococcus aureus* (November 2021, March and November 2022, January and February 2023), *Stenotrophomonas maltophilia* (September 2023) and *Mycobacterium avium* complex (February 2023). Her previous cough swabs had repeatedly cultured *S. aureus *. She had recurring emesis prior to CF respiratory exacerbations. In January 2023, she was admitted, electively, to a regional paediatric CF centre for empirical intravenous antibiotics (ceftazidime and tobramycin) for worsening cough and emesis. Examination revealed widespread crackles and mild intercostal retractions; she was non-coryzal. Her port-a-cath was accessed for antibiotic administration, and within minutes of saline being instilled she developed a pyrexia (38.6 °C) with associated rigours. Thereafter, blood cultures (BACT/ALERT PF Plus) were collected centrally via the port-a-cath and from peripheral venous sites. Empiric peripherally administered ceftazidime (50 mg kg^−1^, 8 hourly) and tobramycin (10 mg kg^−1^, daily) were commenced intravenously as per local CF antimicrobial guidelines.

**Fig. 1.**
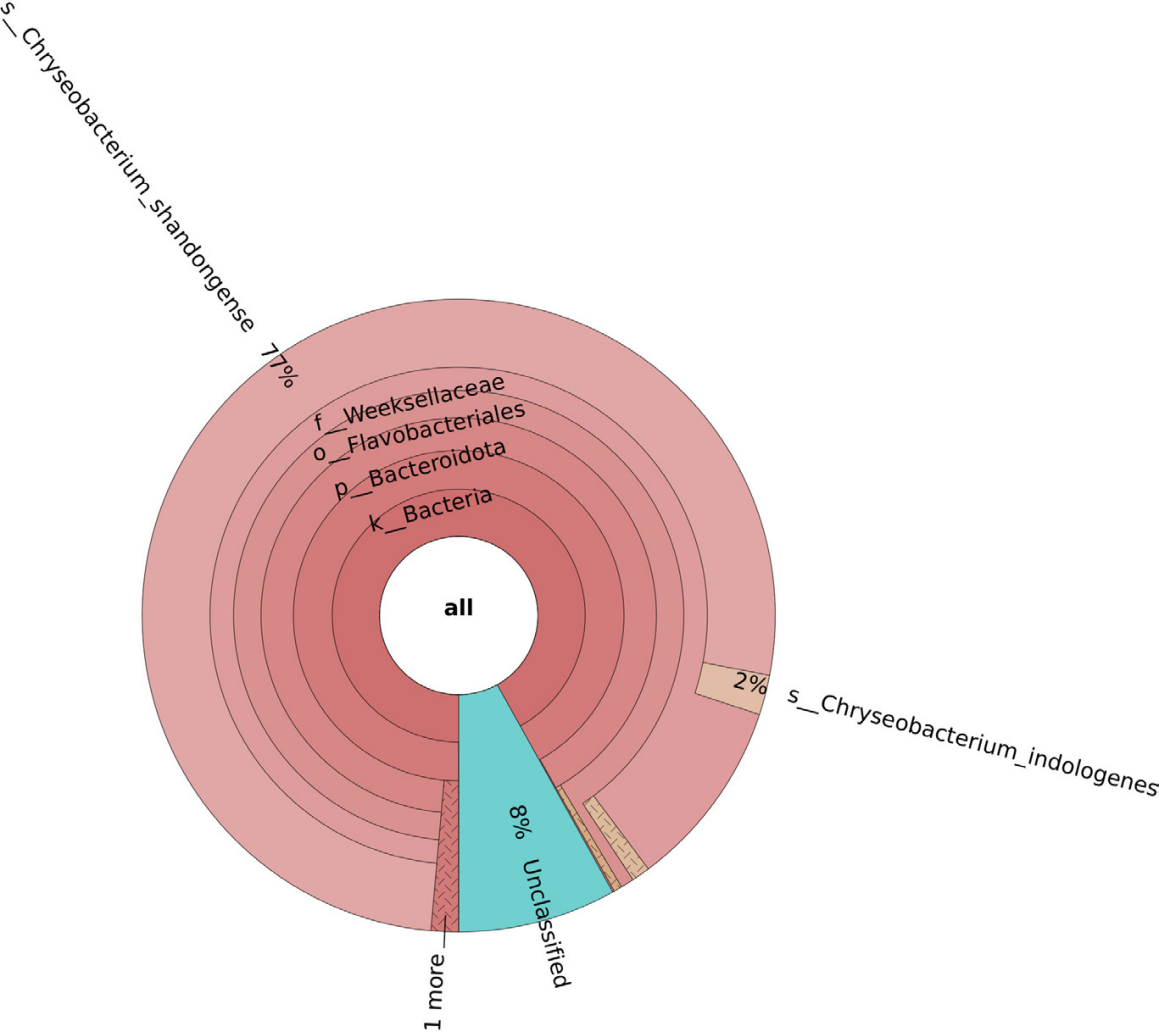
Visualization using the Krona tool of the metagenomic classification of 13 000 reads from the *C. shandongense* isolate, following read assignment by the original BugSeq algorithm.

Following positive growth signal on the bioMérieux BACT/ALERT 3D instrument, the broth was cultured on solid agar plates for 48 h, with blood and MacConkey plates incubated in air at 35–37 °C, blood agar incubated anaerobically at 35–37 °C and chocolate vancomycin agar incubated in 5–10 % CO_2_ at 35–37 %. Following 29 h of incubation at 35–37 ^o^C, the port-a-cath blood cultures returned as positive. Gram-negative bacilli were seen on microscopy. The bacilli were subsequently identified from isolates cultured on nutrient agar as *C. shandongense* by matrix-assisted laser desorption/ionization time-of-flight mass spectrometry (MALDI-TOF MS) using a BrukerMALDI Biotyper with BDAL (library version 12). This was later confirmed using whole-genome sequencing, again identifying the bacilli as *C. shandongense* (Fig. 1).

Following the initial Gram-negative bacilli result, a gentamicin lock (1.5 ml of 9 mg ml^−1^ concentration) was commenced into the port-a-cath, as per the manufacturer’s volume instructions, for 24 h. Repeat blood cultures were collected from the port-a-cath. The peripheral blood cultures remained sterile and no further pyrexia or rigours occurred. The clinical microbiology team were consulted regarding identification and treatment. As the European Committee on Antimicrobial Susceptibility Testing (EUCAST) and Clinical and Laboratory Standards Institute (CLSI) do not publish either breakpoints or consensus epidemiological cut-off (ECOFF) values for *Chryseobacterium* spp., Table 1 presents antimicrobial susceptibility results using EUCAST pharmacokinetics–pharmacodynamics (non-species-related breakpoints). Following a literature review and local availability, 1.5 ml ciprofloxacin locks were instilled using a 0.125 mg ml^−1^ concentration, per the port-a-cath manufacturer’s volume instructions.

**Table 1.** Table demonstrating the antibiotic minimum inhibitory concentrations (MICs) in mg l^−1^ and sensitivity (S) or resistance (R) for *C. shandongense* cultured centrally via the patient’s port-a-cath

Antimicrobial	MIC (observed in mg l^−1^)	Result	S≤	R≥
**Ceftazidime**	2	S	4	8
**Ciprofloxacin**	0.25	S	0.25	0.5
**Gentamicin**	3	R	0.5	0.5
**Tobramycin**	96	R	0.5	0.5
**Doxycycline**	3	–	–	–

–, no result/no criteria.

Repeated port-a-cath blood culture, with appropriate discard of instilled antibiotic lock, continued to yield Gram-negative bacilli at 16 h of incubation (45 h from admission), again subsequently identified as *C. shandongense *. The patient’s sputum was sent for microbiological analysis and *Chryseobacterium* spp. were not cultured. Swabs sent for culture of her nebulizer device were sterile. Investigations on the day of presentation returned a white cell count of 12.8×10^9^ (range 5–12×10^9^), and a C-reactive protein of 21 mg l^−1^ (normal 0–5 mg l^−1^). As she remained systemically well, this case was likely an isolated port-a-cath infection without peripheral bacteraemia. Colonization of the line without infection is also possible, but no alternative cause for her pyrexia was identified. Typically, this patient does not develop pyrexia during pulmonary exacerbations of CF. The results were further discussed with the clinical microbiology team. Subsequent paired surveillance blood cultures following 3 and 7 days of antibiotic locks remained sterile. The ciprofloxacin locks were administered for a total of 2 weeks. The patient remained otherwise well and apyrexial throughout the admission. A follow-up port-a-cath blood culture at 6 and 12 weeks post-discharge, at the time of her subsequent routine admissions, yielded no growth and thus successful eradication.

## Discussion

There are no descriptions of pathogenic manifestations of C. *shandongense* cited in the literature to date. We report the first documented case of successful eradication of a *C. shandongense* intravascular bloodstream infection involving a port-a-cath. *Chryseobacterium *-contaminated surgically implanted devices such as central intravenous lines and prosthetic valves have been documented [3]. *Chryseobacterium indologenes *, which shares the same genus, has been shown to cause severe, invasive infections, especially in patients with risk factors such as indwelling devices, immunosuppression and a history of prolonged courses of broad-spectrum antibiotics [3]. The prevalence of *C. indologenes* has been increasing in recent years, and infections have been associated with an overall mortality rate, in small case series (*n*=16), of 17–28 % [4]. This series showed 63.6 and 35.2% mortality rates in those patients specifically with *C. indologenes* bacteraemia and pneumonia, respectively [4]. Patients with CF potentially have multiple risk factors, such as indwelling devices, which could put them at greater risk for *Chryseobacterium* species-associated infection. Therefore, recognition of this bacterium and its subsequent treatment are essential for reducing mortality risk. Sputum isolates of *Chryseobacterium* spp. from patients with CF have been demonstrated [5, 6]. Additionally, when detected in sputum samples, *Chryseobacterium* spp. were not associated with a decline in pulmonary function and mortality [5, 6]. Considering the limited data available regarding *Chryseobacterium* spp., further studies are essential to fully understand their clinical significance in CF.

Notably, *Chryseobacterium* spp. are reported to be multidrug-resistant organisms, posing challenges for antibiotic choice and treatment options [7]. Additionally, their intrinsic multidrug resistance contributes to the high mortality, as described above. The agents typically deployed to treat non-fermenting bacteria are expected to remain effective, such as meropenem, ciprofloxacin, aminoglycosides and piperacillin–tazobactam. Information regarding *Chryseobacterium* susceptibilities *in vitro* remains relatively sparse, as the species has rarely been isolated clinically [8, 9]. Literature reports suggest that the species is frequently resistant to a wide range of antibiotics, including penicillins, first-, second- and third-generation cephalosporins, and monobactams such as aztreonam. The limited literature available at the time of this report suggests that the most effective antibiotics are quinolones and trimethoprim–sulfamethoxazole, with 95 % of isolates susceptible [9]. Duration of therapy in most cases is 7–14 days. Ultimately, treatment should be tailored with results of antibiotic susceptibility tests and assessment of the patient’s clinical response. In this case, we have demonstrated that effective eradication from a port-a-cath can be achieved with appropriate antibiotic locks through the use of both gentamicin (for 24 h) and ciprofloxacin for 2 weeks. Additionally, salvage as opposed to removal of the port-a-cath was attempted due to prior difficult access and an ongoing requirement for regular intravenous antibiotics.

Herein, we demonstrated successful eradication of *C. shandongense* from a port-a-cath device, including the MICs required in this case. The result was eradication of central access infection, preventing progression to bacteraemia/septicaemia and preserving central access in a child with CF and established respiratory disease.
